# Technological Features of Immersive Virtual Reality Systems for Upper Limb Stroke Rehabilitation: A Systematic Review

**DOI:** 10.3390/s24113546

**Published:** 2024-05-31

**Authors:** Chala Diriba Kenea, Teklu Gemechu Abessa, Dheeraj Lamba, Bruno Bonnechère

**Affiliations:** 1 Department of Information Science, Faculty of Computing and Informatics, Jimma Institute of Technology, Jimma University, Jimma P.O. Box 378, Oromia, Ethiopia; 2 REVAL Rehabilitation Research Center, Technology-Supported and Data-Driven Rehabilitation, Data Science Institute, Faculty of Rehabilitation Sciences, University of Hasselt, 3590 Diepenbeek, Belgium; teklugem@yahoo.com (T.G.A.); bruno.bonnechere@uhasselt.be (B.B.); 3 Department of Special Needs & Inclusive Education, Jimma University, Jimma P.O. Box 378, Oromia, Ethiopia; 4 Department of Physiotherapy, Faculty of Medical Sciences, Institute of Health, Jimma University, Jimma P.O. Box 378, Oromia, Ethiopia; dheeraj.ramesh@ju.edu.et; 5 Technology-Supported and Data-Driven Rehabilitation, Data Sciences Institute, Hasselt University, 3590 Diepenbeek, Belgium; 6 Department of PXL—Healthcare, PXL University of Applied Sciences and Arts, 3500 Hasselt, Belgium

**Keywords:** immersive virtual reality, development, validation, stroke, upper extremities, rehabilitation technology

## Abstract

Stroke is the second most common cause of death worldwide, and it greatly impacts the quality of life for survivors by causing impairments in their upper limbs. Due to the difficulties in accessing rehabilitation services, immersive virtual reality (IVR) is an interesting approach to improve the availability of rehabilitation services. This systematic review evaluates the technological characteristics of IVR systems used in the rehabilitation of upper limb stroke patients. Twenty-five publications were included. Various technical aspects such as game engines, programming languages, headsets, platforms, game genres, and technical evaluation were extracted from these papers. Unity 3D and C# are the primary tools for creating IVR apps, while the Oculus Quest (Meta Platforms Technologies, Menlo Park, CA, USA) is the most often used headset. The majority of systems are created specifically for rehabilitation purposes rather than being readily available for purchase (i.e., commercial games). The analysis also highlights key areas for future research, such as game assessment, the combination of hardware and software, and the potential integration incorporation of biofeedback sensors. The study highlights the significance of technological progress in improving the effectiveness and user-friendliness of IVR. It calls for additional research to fully exploit IVR’s potential in enhancing stroke rehabilitation results.

## 1. Introduction

Stroke is a leading cause of death and long-term disability worldwide, responsible for approximately 12% of all deaths [1]. It ranks as the second most common cause of death globally [2]. The incidence of stroke is escalating in both developed and developing countries, presenting a global health challenge [3].

At the onset of a stroke, survivors experience a wide array of symptoms, including motor, mental, lingual, sensory, and cognitive impairments. These impairments lead to substantial functional challenges in daily life and a decreased quality of life. Upper limb impairments are the most prevalent consequence, and the more disabling post-stroke conditions [4]. Rehabilitating upper limb function is critical for stroke patients, significantly affecting their perceived disability and quality of life [5].

However, access to rehabilitation services is often limited, which is a significant barrier to recovery identified by the World Health Organization [6]. It is, therefore, crucial to develop innovative solutions to enhance the availability of rehabilitation services for stroke survivors. The rapid advancements in technology and computer science have significantly altered our environment and lifestyle, introducing new technologies to augment physiotherapy and rehabilitation processes, termed technology-supported rehabilitation [7].

One technology in particular, the Virtual Reality (VR), is becoming increasingly tested and implemented in the healthcare sector. This popularity has increased significantly in the past decade, with a particularly rapid acceleration since 2016. This spike can be attributed to the production of affordable and user-friendly VR hardware by various firms, which has made the technology more accessible to a wider range of people [8]. It is a three-dimensional computer-generated simulated environment, which attempts to replicate real world or imaginary environments and interactions, thereby supporting work, education, recreation, and health [9].

Immersive Virtual Reality (IVR) is one of the computer applications used to visualize virtual environments, including their surrounding objects. It also allows us to observe, listen to the sounds around us, and interact with people in virtual environments by acting like we are in the real world [10,11]. IVR provides an opportunity to create a sense of immersion, simulating the feeling of being in the real world. However, this immersion varies across different degrees, leading to the categorization of VR into three main types: non- IVR, semi-IVR, and fully IVR.

In non-IVR, users engage with virtual environments via traditional displays, such as smartphone screens or computer monitors, without a sense of physical presence within the virtual world. Semi-IVR facilitates interaction with virtual content while allowing users to remain cognizant of the real world, often employing large (incurved) screens or projection systems. Fully IVR provides an experience wherein the user perceives themselves to be present within the virtual environment, typically with minimal or no awareness of their actual physical surroundings, primarily through the utilization of head-mounted displays [12].

Within VR setups, users primarily engage with the virtual environment via various input devices such as controllers, joysticks, or motion capture cameras. Thanks to technological developments, VR is becoming more and more used in diverse sectors such as healthcare, education, entertainment, military, etc. In healthcare, IVR can be used for training medical professionals, pain management, treating mental health conditions, and rehabilitation. IVR for rehabilitation is an emerging field, experiencing rapid development and research progress.

Recent research has highlighted multiple benefits of IVR in rehabilitation, including for example, increased engagement and motivation, immediate feedback, improved patient outcomes, 3D motions and activities mimicking daily living activities, and customizable dual or triple tasks [13,14]. Compared to non-IVR, IVR not only enhances motivation and adherence [15] to treatment but also offers greater clinical efficacy [16]. This improvement is attributed to the immersive experience and sense of presence provided by IVR, which facilitate more challenging dual-task scenarios and elevated cognitive stimulation. Consequently, these factors lead to increased brain activation during rehabilitation exercises, as suggested by current evidence [17].

Consequently, IVR is currently being applied across a broad spectrum of rehabilitation areas, encompassing neurological rehabilitation for postural balance [18], motor functions [19,20], physical therapy [21,22], and cognitive rehabilitation [19,23]. Additionally, it has shown potential for providing pain relief compared to conventional therapy in various pathologies [24]. IVR emerges thus as a promising tool in the healthcare sector and more particularly in rehabilitation.

IVR interventions have proven effective in stroke rehabilitation [25]. Studies indicate significant improvements in the upper limb function of stroke patients, highlighting its potential as a therapeutic tool in rehabilitation and neurorehabilitation fields [26]. The immersive and interactive nature of IVR facilitates the creation of controlled environments that support intensive, repetitive, and task-oriented practice, offering advantages over conventional therapy.

The adoption of technology-supported rehabilitation, such as IVR, is also increasingly being used for its capacity to provide objective assessments that can be automated, thereby saving time and facilitating the precise evaluation of motor functions [7]. This approach may take into consideration various patient-specific factors, including kinematics, activity levels, intensity of movements, muscle activity, co-contraction patterns, posture, motion fluidity during exercises, heart rate, and stress levels, among others. It also allows for tracking adherence to therapy. IVR’s immersive nature not only enhances patient engagement but also supports adjustments related to the body (for instance, alterations in the size and length of body segments and body composition), the environment, and social interactions. A notable feature of IVR is its ability to capture a wide range of measurements, referred to as biomarkers, during rehabilitation exercises [27]. Defined by Wagner, rehabilomics is an innovative approach that discusses biomarkers within research and clinical contexts to address the gaps and needs of clinical treatments specific to physical medicine and rehabilitation [28]. This concept merges the systematic gathering of data on phenotypes relevant to rehabilitation and a multidisciplinary analysis of biomarkers, aiming to deepen our understanding of the biological aspects, functionality, prognosis, complications, treatment options, adaptation processes, and recovery in individuals with disabilities [29].

Specifically, IVR can monitor the mobility and function of the upper limbs during the rehabilitation exercises without the necessity for external sensors, making it a less intrusive and more user-friendly option for both patients and clinicians [24,25]. Additionally, IVR systems have the capability to gather more physiological data, including autonomic responses, through techniques such as pupillometry to inform about pain and stress level of the patients [30].

Despite these advancements, the application of IVR in the rehabilitation of upper limb stroke both from a rehabilitation perspective and from the assessment (i.e., rehabilomics) remains an area that requires further research.

Therefore, this study aims to systematically review current literature in this domain to outline the technical aspects of IVR systems from both software and hardware perspectives. This study will also discuss current limitations and future challenges, contributing valuable insights into the potential and further development of IVR for stroke rehabilitation.

## 2. Materials and Methods

The review protocol has been registered on the Open Science Framework (OSF) [31].

### 2.1. Search Strategy

The Preferred Reporting Items for Systematic Reviews and Meta-Analyses (PRISMA) was used [32]. A predefined search strategy was employed to search Scopus, PubMed, and Web of Science databases for relevant papers (last update: 20 March 2024). In total, 154 papers were retrieved, with 28 from PubMed, 72 from Web of Science, and 54 from Scopus (Table 1).

### 2.2. Study Selection

The review adhered to the PRISMA guidelines to ensure strictness, replicability, reliability, and accuracy. Initially, one reviewer conducted searches on the PubMed, Web of Science, and Scopus platforms using the defined search strategy. Subsequently, the retrieved papers were imported into Zotero to eliminate duplicate results. All references and citation details from the chosen electronic databases were consolidated and exported from Zotero 6.0.26 into Rayyan, version 6.0.26.

The authors systematically screened and selected papers through a three-step process involving titles, abstracts, and full-text screening, utilizing Rayyan. The first author screened papers based on titles, followed by independent abstract screening by two screeners. Full-text screening was then conducted by the last two screeners. This three-step process ensured screening reliability, with the inclusion of relevant papers determined by the consensus of both screeners. During the screening process, we applied various inclusion and exclusion criteria. There were no limitations on the publication year of articles; however, only English-language articles published in peer-reviewed journals were considered for inclusion. The selection focused solely on stroke, excluding articles on other diseases causing upper limb impairment. Non- and semi-IVR, as well as review papers, were excluded.

### 2.3. Data Extraction

The data extraction process involved the use of the following parameters: country, methodology adopted, software used, virtual reality headset, platforms (standalone IVR headset, mobile, tablet, laptop, or desktop), game types (open source, commercial, custom, or developed), IVR game scenarios, technical evaluation, and clinical evaluation. To ensure reliability, the two researchers worked independently to extract the data. To ease the visualization of the different parameters of the included studies (i.e., type of IVR, localization), we used network analysis to plot the results. The distribution of the available evidence was assessed using a network geometry graph in which the width of the continuous line connecting nodes corresponding to the number of trials directly comparing the interventions [33].

### 2.4. Quality Assessment

We evaluated the quality of each study included in the review by utilizing the Critical Appraisal Skills Program (CASP) Qualitative Studies Checklist [34]. The CASP was assessed by two researchers.

## 3. Results

**Figure 1:**
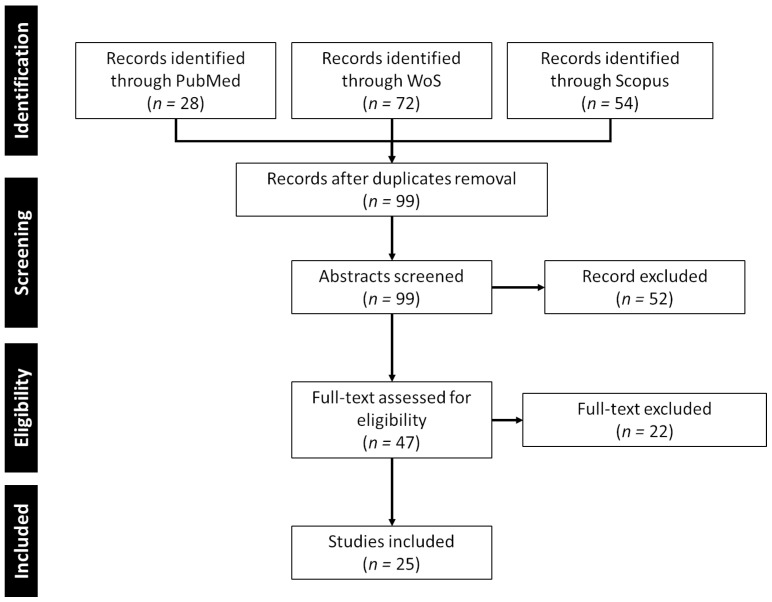
PRISMA flow diagram of study selection.

In total, 159 papers were screened following the PRISMA guidelines, and 25 were finally included in this review (Figure 1).

### 3.1. Quality Assessment

**Figure 2:**
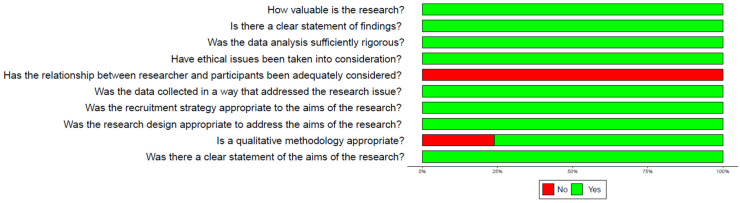
Overall quality of the studies (CASP assessment).

The CASP assessment results revealed that the mean score of individual studies was 8.9 out of 10. This outcome affirms the relevance and appropriateness of the papers for the study. However, it is noteworthy that the relationship between researchers and participants was never explicitly stated in the included studies (Figure 2).

### 3.2. Patients and Interventions

In total, 566 participants were included in this review: 337 patients in the intervention group and 229 controls. The mean age was 53.8 (standard deviation: 10.4) years old. There were more males than females (58.62%). Most of the studies were performed in the chronic phase (*n* = 16, 64%), or subacute (*n* = 6, 24%); note that some studies included patients in various phases.

**Figure 3:**
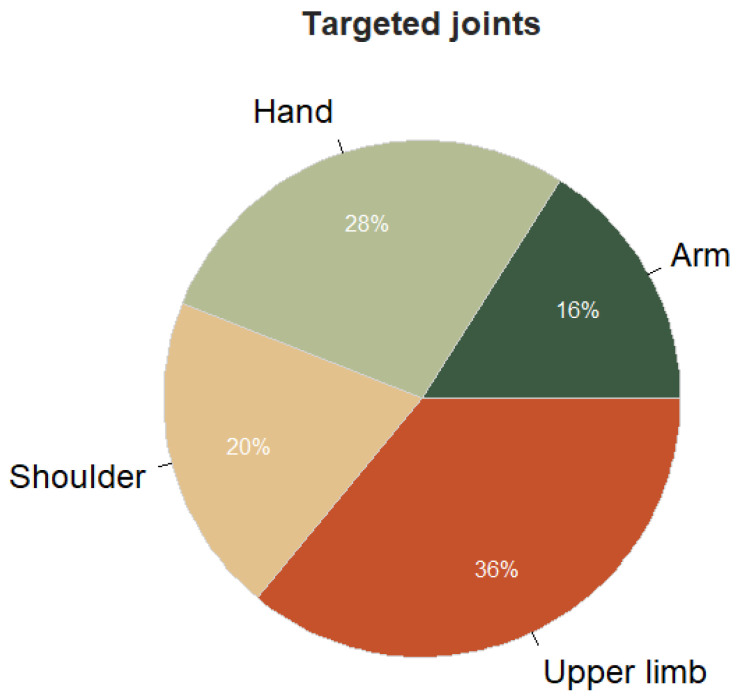
Most frequent targeted joints in the included IVR systems.

Concerning the intervention, the median duration was 3 weeks [p25 = 2; p75 = 4.75], with a median of 3 [1 ; 5] sessions per week with a median duration of 30 [25 ; 45] minutes. The vast majority of the studies were performed in the hospital, with only one study being performed at home [5]. As presented in Figure 3, most of the studies are focusing on the upper limb globally (including mostly shoulder, elbow, and wrist mobility), but there are also a significant number of systems focusing only on the hand function.

Complete characteristics of the patients are presented in Table 2.

### 3.3. Country: IVR Games Developed and Validated

The findings of the review revealed that in all selected studies, the countries where IVR games for ULSR were developed and validated were the same. The studies were mostly conducted in Korea (*n* = 4, 16%), the USA (*n* = 3, 12%), Spain (*n* = 3, 12%), China (*n* = 3, 12%), Taiwan (*n* = 3, 12%), Belgium (*n* = 2, 8%), the UK (*n* = 2, 8%), and in Italy, India, Brazil, Poland, and Germany.

### 3.4. Software

The common software-related technological aspects of IVR games include game engines and programming languages. The findings of this review show that the Unity 3D engine (*n* = 13, 52%) was the predominant game engine used, with Unreal Engine (*n* = 2, 8%) being the second most employed.

The C# was the most extensively utilized programming language, while Java script was mentioned in only one paper. Notably, the Unity 3D engine was frequently paired with the C# programming language.

The finding pointed out that most of the systems (*n* = 19, 76%) were custom-developed, either from scratch or by customizing already-developed games, while only (*n* = 2, 8%) studies were performed with commercial games and (*n* = 1, 4%) with open-source games. However, (*n* = 3, 12%) of the studies did not specify the type of games used (see Table 3 for complete results).

Table 4 presents a comprehensive overview of the main game scenarios now used in rehabilitative environments, categorized according to the specific skills they aim to improve. These scenarios encompass a variety of exercises that target both fine motor skills and exact manipulations, as well as exercises that aim to enhance gross motor abilities and general body mobility. In addition, the table outlines the technological features of these games, emphasizing the use of VR combined with motion tracking and real-time feedback mechanisms that enable these therapeutic interventions. The current overview seeks to offer a thorough understanding of how various games contribute to distinct rehabilitation objectives. The methodology adopted for the development of IVR games for ULSR was explicitly mentioned in only two papers screened. They are the VR2 clinical study design and user center design. There are three phases of VR clinical study designs. The VR2 study concentrates on assessing feasibility, acceptability, tolerability, and initial clinical efficacy, while in user-centered design (UCD) methodology, designers consider users’ demands at every stage of the IVR game design process. To produce an IVR game that is highly usable and accessible for users, UCD design teams incorporate users throughout the entire design process. This design approach is user targeted.

### 3.5. Hardware

First, concerning the headsets, our results show that Oculus Quest (*n* = 12, 48%), HTC Vive (*n* = 7, 28%), and Oculus Rift (*n* = 2, 8%) were the most used system. Note that for three studies, the type of headset was not specified.

The study’s findings revealed that IVR games for ULSR were primarily played on standalone headsets (*n* = 8, 32%), desktops (*n* = 8, 32%), laptops (*n* = 3, 12%), and mobile devices 1 (*n* = 1, 4%). Again, here, we noticed that in five studies (*n* = 5, 20%), the type of platform was not specified.

**Figure 4:**
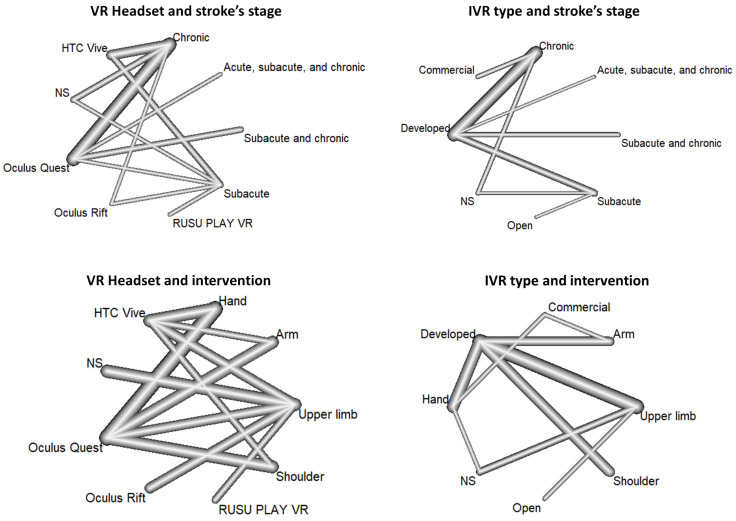
Network of investigated associations between VR headset, IVR type, stroke’s stage, and intervention. The line width corresponds to the number of patients directly associating the different modalities.

To better visualize the relationship between the different headsets commonly used and the type of IVR solutions (i.e., specially develop, commercial or open access), we performed network analysis with the stroke’s stage and the targeted localization. Results are presented in Figure 4. Concerning the headset, as already seen, the most common in the Oculus Quest used in the chronic phase, targeting the hand and arm function. Concerning the type of IVR the most commonly used are specifically developed ones, also used in the chronic phase to target upper limb function globally. 

### 3.6. Technical Evaluation

In this review, none of the IVR games utilized for ULSR were evaluated by the game developers and/or rehabilitation specialists by using different game evaluation parameters, commonly used to evaluated system, such as the graphic rendering of games, quality and appropriateness of audio used, haptic feedback, use of biofeedback sensors, whether or not there can be customizable environments for different level patients, a scoring system, levels and progression, virtual rewards and incentives, and motion tracking.

## 4. Discussion

The aim of this review was to summarize the technologies currently being used to create IVR rehabilitation. These technical features include game engine, programming language, adopted methodology, headsets, platform, technical evaluation, and clinical validation. The specifics of each discussion are outlined below.

### 4.1. Game Engine and Programming Language

The study results emphasize that C# is the most widely used programming language for developing applications for IVR serious games. Furthermore, the C# programming language is the most adaptable programming language for creating IVR apps [59] and for developing IVR games with the Unity 3D game engine [60]. The Unity 3D game engine is widely recognized as the most used tool for IVR game development. This point is further supported by another study showing that Unity 3D is a powerful tool enabling programmers to create IVR game applications [61,62]. Unity simplifies the process of developing VR applications for popular operating systems such as Windows, iOS, and Android, as well as for most top gaming consoles and the web. Furthermore, it is available in both free and professional licenses, facilitating quick prototyping and the distribution of created applications across various IVR platforms.

### 4.2. Methodologies Adopted in IVR Games

One striking result of this study was that the adopted methodology was only outlined in two papers. They are VR2 clinical study design [57] and user-centered design [44]. There are three phases to the VR clinical trial designs [63]: VR1, VR2, and VR3. The production of content using the principles of human-centered design in collaboration with patients and providers is the main emphasis of VR1 studies. Early testing in VR2 trials is focused on first clinical efficacy, acceptability, tolerability, and feasibility. Randomized controlled trials, or VR3 trials, assess effectiveness in comparison to a control group.

User-centered design, on the other hand, is an iterative design approach where designers prioritize users and their needs at every stage of the design process. But there are methodologies that can be adopted in the development of IVR games, such as experience design [64], iterative design [65,66], experimental design [67], rapid prototyping methodology [68], and participatory design or co-design [69,70]. Participatory design, which involves professionals from different disciplines, has been employed by many scholars in healthcare for IVR game development [71,72] and could be particularly relevant for the development of rehabilitation solutions but has not been investigated yet in this particular field.

### 4.3. Types of IVR Headsets

Various companies have developed different headsets, including but not limited to Oculus Quest, HTC Vive, Apple Vision Pro, HoloLens, and Google Cardboard. However, in this review, only four distinct IVR headsets were used, namely the Oculus Quest (*n* = 12, 48%), HTC Vive (*n* = 7, 28%), Oculus Rift (*n* = 2, 8%), and Rusu Play VR (*n* = 1, 4%). These results are confirmed by different other studies showing that the Oculus Quest and HTC Vive are among the most popular IVR headsets [73,74]. Both of these systems have pros and cons. The Oculus Quest is a standalone device, eliminating the need for a connection to a computer or mobile phone. Moreover, it facilitates casting to a TV or smartphone and supports wireless streaming from a PC via Air Link or Virtual Desktop. Conversely, the HTC Vive requires additional hardware (computer with good graphical card) or software and is restricted to the SteamVR library. While the Oculus Quest is recognized for its convenience, versatility, and affordability, the HTC Vive is lauded for its power, immersion, and customization capabilities. Notably, many scholars have used the Oculus Quest in several studies focusing on shoulder, hand, balance, and arm motor rehabilitation [75,76,77].

### 4.4. Platform for Implement IVR for ULSR

IVR platforms encompass devices that enable users to immerse themselves in and interact with virtual environments in a realistic and engaging manner. Various types of immersive VR platforms exist, differing in hardware and software requirements, level of immersion, and content availability. Common types of platforms include standalone, desktop, laptop, and smartphone.

Standalone VR utilizes a standalone VR headset (e.g., Oculus Quest 2, Meta Quest 2 without Link Cable, and Meta Quest Pro) with its own processing power, graphics, storage, battery, speakers, cameras, and sensors for the IVR experience. Different studies also support the idea that standalone VR is the most commonly used and effective platform [78,79,80].

Desktop VR involves using a desktop computer paired with a tethered VR headset (such as the HTC Vive) to run VR applications. In this study, desktop VR accounted for (*n* = 8, 32%) of the usage which also its suitability was mentioned in different papers [73], while laptops and smartphones were not much utilized.

### 4.5. IVR Game Types

IVR games can be effectively employed by ULSR, offering enjoyable and motivating exercises. Depending on their purpose, IVR games for stroke rehabilitation can be classified as open, commercial, or developed. The study’s findings revealed that the majority of IVR games for ULSR were specific solutions (*n* = 19, 76%) rather than commercially available solutions or open source. This result also supported the fact that in several studies, they used their own games by developing them from the scratch [61,81]. Such types of solutions are of course more adapted to the patients and the rehabilitation process but are more costly to develop and maintain in comparison with commercially available solutions.

### 4.6. IVR Game Evaluation

As presented in the result section, the IVR games used for ULSR were not evaluated by game developers or rehabilitation professionals while multiple studies recommended the need for serious game evaluation before using it, since it is for a specific purpose [82,83,84]. There are several aspects of game evaluation, such as graphic rendering of games, quality and appropriateness of audio used, haptic feedback, use of biofeedback sensors, whether there can be customizable environments for different level patients, a scoring system, levels and progression, and virtual rewards and incentives. These aspects have more detail elements; for instance, graphics rendering has dimensionality, perspective, color, presentation, and realism [85].

The audio system can contribute to a more immersive experience in games played on screens [86]. The haptic feedback has been shown to enhance user performance and enhance interaction in a fully IVR environment [87,88]. In addition, haptic feedback may support immersion and presence in IVR environments [89]. Moreover, the use of biofeedback sensors within IVR environments has also been stated in different studies that it can improve user performance [90,91].

Furthermore, in relation to whether there can be customizable environments for different levels of patients, a scoring system with levels and progression, virtual rewards and incentives, and motion tracking, much needs to be evaluated before IVR game utilization.

### 4.7. Technical Implications

Technological advancements in IVR encompass both hardware and software. The evidence demonstrates a consistent increase in the development and utilization of IVR for ULSR over the years. The degree of immersion has also progressed from non-IVR to semi-IVR and now fully IVR, owing to enhancements in VR headsets, controllers, powerful computers, and game engine technologies.

This evolution presents opportunities for further improvement in technological aspects, such as immersion level, state of engagement and motivation, graphical enhancements, simplified immersive game development, a general framework for development and validation, headsets without controllers, feedback mechanisms, and notifications. Additionally, the study suggests that widely adopted elements in the realm of IVR for ULSR include the Unity 3D game engine, C# programming language, preference for developed games over open or commercial ones, and the utilization of the Oculus Quest standalone headset in terms of technical aspects.

### 4.8. Clinical Implications of Technical Means and Metrics in IVR

The adaptation of IVR to various stages of stroke rehabilitation (acute, subacute, and chronic), as presented in Table 3 and Figure 4, has the potential to greatly impact patient outcomes. For example, in the initial phase of rehabilitation, it may be more advantageous to use systems that provide controlled and gradual movements to avoid muscular tension. In later stages, more demanding tasks can be introduced to assist with muscle rebuilding and neuroplasticity.

Similarly, the differentiation in the implementation of IVR systems for various body parts highlights specific requirements needed to develop specific and adapted IVR systems. For example, the hand rehabilitation process requires a higher level of sensor integration and feedback precision compared to the strengthening of gross motor abilities in the upper limb. This level of precision has the potential to result in more focused and efficient rehabilitation protocols, which are essential for addressing the intricate requirements of stroke recovery.

Our findings have an important clinical implication regarding the criteria used to assess the quality of rehabilitation. Using the data collected during the rehabilitation process [92] (i.e., speed, ranges of motion, smoothness) offers a quantitative foundation for evaluating patient advancement. These parameters are crucial for evaluating the clinical effectiveness of IVR systems. Furthermore, comprehending these technical aspects aids in enhancing VR applications to more effectively fulfill therapeutic requirements, potentially resulting in personalized rehabilitation plans based on individual progress indicators.

### 4.9. Current Limitations

Various technological factors have been evaluated in this review, but additional inquiries are required before fully apprehending the potential of IVR in rehabilitation. The analysis of the various VR headset types and development environments yields valuable insights into current practices. However, a more significant and insightful understanding can be gained by considering the clinical implications of how these technologies are utilized, taking into account the specific requirements of stroke rehabilitation stages and targeted limb functionalities. This technique not only meets the requirements of clinical demands but also advances the boundaries of how immersive technology may be used for health benefits. It highlights the need for more technical examination in future VR research in the field of rehabilitation.

Areas that need more consideration encompass the degree of immersion and the differences between semi-immersive, IVR and augmented reality, for example, the dimensions and weight of the headset, the use of controllers or marker less camera to track upper limb’s motion, and the potential problem related to VR sickness according to the different game’s scenarios. As we have seen, there are different options to integrate IVR into care. These include developing and testing different games specifically designed for this purpose, which is currently and by far the most commonly used practice or used commercially available solutions. Nevertheless, in both cases, it is essential to conduct a comprehensive examination of the technological components, encompassing the stages of development, validation, and customization for various contexts. This analysis should take into account crucial elements such as motivation [93,94,95], flow [96], usability [39,44], skill [97], judgment [98], technological adoption and acceptance [99,100,101,102], safety and comfort [46], satisfaction [39,44,45,46,94], immersion [103], and sense of presence and emotion [104].

Another crucial aspect requiring further investigation is the utilization of data gathered during the rehabilitation process (i.e., rehabilomics). These data are essential not only for analyzing patient progress [25] but also for automatically adjusting the settings of rehabilitation exercises [7]. Despite their clear potential and added value [105], systems that comprise both hardware and software components must undergo comprehensive validation before they are employed in clinical assessments.

This requirement might be ascribed to the newness and continuous use of IVR games for ULSR. Furthermore, the research has not yet examined the use of IVR for ULSR in low- or middle-income countries, despite the increasing number of stroke patients in developing areas. The majority of the reported studies have been conducted in high-income countries.

## 5. Conclusions

This systematic review provided a comprehensive analysis of the technological aspects of IVR systems for ULSR. Through examining 25 different systems, this study highlights Unity 3D and C# as the predominant tools for developing immersive applications, with Oculus Quest being the most used headset. Another important finding is that most of the systems used have been specifically developed, highlighting future challenges in sustainably implementing such systems in daily care. Despite significant advancements in IVR technology, which include customizable environments, we identified several areas needing further exploration such as the validation of game scenarios, the choice of the best combination of hardware and software, and the integration of biofeedback sensors. Overall, these findings suggest a promising future for IVR in rehabilitation, urging continued innovation and research to enhance the efficacy and user-friendliness of these systems. The expansion of technological capabilities in IVR is crucial for advancing rehabilitation practices and improving patient outcomes in stroke recovery.

## Figures and Tables

**Table 1:** Systematic review sources: search databases, strings, and numbers of results.

Databases	Strings	Numbers Results
PubMed	(((“immersive virtual reality”[Title/Abstract]) AND (“upper limb”[Title/Abstract] OR “upper extremity”[MeSH Terms])) AND (stroke[Title/Abstract])) AND (rehabilitat*[Title/Abstract])	28
Web of Science	TI = ((“immersive virtual reality” OR “serious gam*”) AND (“cyber*”)) and TS = ((“immersive virtual reality” OR “serious gam*”) AND (“cyber*”))	72
Scopus	TITLE-ABS (“immersive virtual reality” OR “serious gam*”) AND (“cyber*)	54

**Table 2:** Characteristics of the patients.

Study	Participants		Age (Mean)	Stroke Stage	Target	Setting	Duration of the Intervention (Week)	Number of Sessions Per Week	Duration of One Session (Minute)	Clinical Evaluation
	Intervention	Control								
Burton et al., 2022 [35]	25	30	60	Acute, subacute, and chronic	Hand	Hospital	2	NS	NS	ARAT, SUS
Chen et al., 2023 [36]	25	25	58	Subacute	Upper limb	Hospital	2	6	30	FMA-UE
Crepo et al., 2023 [37]	21	NS	59	Chronic	Shoulder	Hospital	1	1	15	ROM
Elor et al., 2018 [38]	6	NS	26	Chronic	Arm	Hospital	1	1	5	Questionnaire
Elor et al., 2022 [21]	5	5	25	Chronic	Shoulder	Hospital	8	2	45	ROM
Everard et al., 2022 [39]	22	23	64	Subacute and chronic	Hand	Hospital	1	1	45	BBT
Fregna et al., 2022 [40]	16	NS	62	Subacute and chronic	Hand	Hospital	1	1	50	FMA-UE
Huang et al., 2022 [41]	18	17	55	Chronic	Upper limb	Hospital	9	3	30	FMA_UE
Huang et al., 2023 [42]	18	17	64	Subacute	Upper limb	Hospital	3	5	30	FMA-UE, BI
Hsu et al., 2022 [43]	15	15	55	Chronic	Upper limb	Hospital	5	2.67	60	FMA-UE
Juan et al., 2022 [44]	14	NS	41	Chronic	Hand	Hospital	NS	NS	NS	LMS
Kamatchi et al., 2023 [45]	8	8	57	Subacute	Upper limb	Hospital	8	5	45	FMA-UE
Lee et al., 2020 [46]	12	NS	40	Chronic	Hand	Hospital	3	2.5	30	ARAT
Lim et al., 2020 [47]	10	10	60	Chronic	Hand	Hospital	4	4	30	BBT, ARAT
Lin et al., 2020 [48]	9	9	22	Chronic	Upper limb	Hospital	2	2	45	FMA-UE
Matamala-Gomez et al., 2022 [49]	20	NS	60	Chronic	Arm	Hospital	5	3	20	FMA-UE, DASH, ROM
Mekbib et al., 2021 [50]	11	12	55	Subacute	Upper limb	Hospital	2	4	60	BI, FMA-UE
Ogun et al., 2019 [51]	33	32	61	Chronic	Upper limb	Hospital	6	3	60	FMA-UE, ARAT
Park et al., 2021 [52]	1	NS	56	Subacute	Hand	Hospital	4	5	20	TULIA
Phelan et al., 2021 [53]	10	NS	11	Chronic	Upper limb	Hospital	1	1	15	ROM
Phelan et al., 2023 [5]	8	NS	13	Chronic	Shoulder	Home-Based	NS	NS	NS	ROM
Sip et al., 2022 [54]	10	10	57	Subacute	Upper limb	Hospital	3	6	30	FMA-UE
Song and Lee 2021 [55]	6	6	64	Chronic	Arm	Hospital	4	5	30	EMG and MFT
Tokgöz et al., 2023 [56]	4	NS	NS	Chronic	Shoulder	Hospital	3	NS	30	ROM
Tran et al., 2021 [57]	10	10	49	Chronic	Arm	Hospital	4	7	30	ARAT

ARAT: Action Research Arm Test; BBT: Box and Block Test; BI: Barthel Index; DASH: Disability of Arm-Shoulder-Hand; EMG: Electromyography; FMA-UE: Fugl-Meyer Assessment for Upper Extremity; LMS: Leap Motion Sensor; MFT: Manual Function Test; NS: Not Specified; ROM: Range of Motion; TULIA: Test of Upper Limb Apraxia; SUS: System Usability Scale.

**Table 3:** Technological aspects of IVR systems.

Study	Country	Methodology Adopted for Development	Game Engine	Programming Language	VR Headset	Platform	Game Types
Burton et al., 2022 [35]	Belgium	NS	Unity 3D	C#	Oculus Quest	Standalone Headset	Developed
Chen et al., 2023 [36]	China	NS	Unity 3D	NS	NS	NS	Developed
Crepo et al., 2023 [37]	Spain	NS	Unity 3D	NS	Oculus Quest	Standalone headset	Developed
Elor et al., 2018 [38]	USA	User center design	Unity 3D	C# and Javascript	HTC Vive	Desktop	Developed
Elor et al., 2022 [21]	USA	NS	Unity 3D	NS	HTC Vive	Desktop	Developed
Everard et al., 2022 [39]	Belgium	NS	Unity 3D	C#	Oculus Quest	Standalone Headset	Developed
Fregna et al., 2022 [40]	Italy	NS	Unity 3D	C#	Oculus Quest	Standalone Headset	Developed
Huang et al., 2022 [41]	Taiwan	NS	NA	NS	HTC Vive	Desktop	Developed
Huang et al., 2023 [42]	Taiwan	NS	Unity 3D	NS	Oculus Rift	Laptop	Developed
Hsu et al., 2022 [43]	China	NS	NS	NS	NS	NS	NS
Juan et al., 2022 [44]	Spain	NS	Unity 3D	C#	Oculus Quest	Standalone Headset	Developed
Kamatchi et al., 2023 [45]	India	NS	NS	NS	RUSU PLAY VR	Mobile	Open
Lee et al., 2020 [46]	Korea	NS	NS	NS	HTC Vive	Desktop	Commercial
Lim et al., 2020 [47]	Korea	NS	NS	NS	HTC Vive	Desktop	NS
Lin et al., 2020 [48]	Taiwan	NS	Unity 3D	NS	Oculus Rift	Desktop	Developed
Matamala-Gomez et al., 2022 [49]	Spain	NS	Unity 3D	C#	Oculus Quest	Desktop	Developed
Mekbib et al., 2021 [50]	China	NS	Unity 3D	C#	HTC Vive	Laptop	Developed
Ogun et al., 2019 [51]	Brazil	NS	NS	NS	NS	NS	Developed
Park et al., 2021 [52]	Korea	NS	NS	NS	HTC Vive	Desktop	Developed
Phelan et al., 2021 [53]	UK	NS	Unreal	NS	Oculus Quest	Standalone Headset	Developed
Phelan et al., 2023 [5]	UK	NS	Unreal	NS	Oculus Quest	Standalone Headset	Developed
Sip et al., 2022 [54]	Poland	NS	NS	NS	Oculus Quest	Standalone Headset	NS
Song and Lee 2021 [55]	Korea	NS	NS	NS	Oculus Quest	Laptop	Developed
Tokgöz et al., 2023 [56]	Germany	NS	Unity 3D	C#	Oculus Quest	NS	Developed
Tran et al., 2021 [57]	USA	VR2 clinical study design	NS	NS	Oculus Quest	Desktop	Commercial

NS: Not specified.

**Table 4:** Description of the games’ scenarios.

Category	Game Scenarios	Detailed Description	Technical Details
Fine Motor Skills	Grasp, Grip, Pinch, and Gross Movement [35]	Involves tasks such as lifting, pouring, and pinching various objects. Aimed at improving fine motor skills crucial for daily tasks.	Utilizes high-precision motion tracking to monitor and adapt to the user’s specific motor capabilities.
	Grasping Cube Object [39]	Requires precise manipulation of a cube within a box, simulating real-world object handling, enhancing hand-eye coordination and spatial understanding.	Employs 3D spatial mapping and real-time feedback to ensure accurate hand positioning and movement tracking.
	Hammering, Ball Catch, Cup Pour, Bubble Touch, Xylophone [46]	Engages users in activities that require various precision movements, enhancing dexterity, and coordination. Each activity targets different motor skills from grip strength to touch sensitivity.	Features adaptive difficulty settings and haptic feedback to reinforce proper hand movements.
	Grasping, Transporting, and Releasing Ball [50]	Focuses on the detailed task of moving objects with precision. This game helps in refining motor control and enhancing cognitive planning associated with hand movements.	Incorporates real-time error correction and motion analysis to tailor exercises to patient needs.
Gross Motor Skills and Body Movement	Catching Falling Stars [38]	Players interact with objects descending along various trajectories, which promotes full-body movement and spatial awareness.	Full-body motion capture technology is used to evaluate and enhance body coordination and reflexes.
	Ball in Hole, Cloud Glasses, Rolling Pin [40]	Tasks involve pushing and rolling motions that engage major muscle groups, ideal for restoring gross motor skills and improving physical coordination.	Combines VR environments with physical props to enhance the realism of interactions.
	Climbing [53]	Climbing simulation that involves extensive upper body movement, enhancing strength, and flexibility. Includes safety features to prevent virtual “falls” and encourage risk-free practice.	Dynamic difficulty adjustment and safety algorithms to simulate realistic climbing challenges safely.
Virtual Reality and Full Immersion	Shooting Gallery, Playground, Basketball Court, Boxing Arena, Fencing Hall [58]	A variety of physically interactive VR scenarios ranging from sports to cooking, designed to engage cognitive functions and physical stamina.	Advanced VR systems with immersive audiovisual environments and interactive gameplay mechanics.
	Living Room, Kitchen, Veranda, Convenience Store [55]	Simulates daily life activities within a household, enabling patients to practice routine tasks in a controlled, virtual environment. Helps in cognitive recovery and independence training.	Lifelike VR settings with detailed object interactions to mimic real-life scenarios and movements.
Simulated Daily Activities	Lifting and Eating an Apple [44]	Simulates the action of eating an apple to coordinate arm lifting with mouth movements, useful for patients recovering from upper limb impairments.	Utilizes biomechanical models to simulate realistic arm and hand movements.
	Dressing, Eating, Drinking, Washing, Brushing Teeth, Combing Hair [52]	Activities designed to mimic essential daily tasks, each targeting specific motor and cognitive skills needed for self-care and independence.	Tailored scenarios that adjust in complexity based on the patient’s progress and capabilities.
	Grasping Object Game [49]	Focuses on mental planning and execution of complex hand movements, with visuo-tactile feedback enhancing the sense of touch and motor planning.	Combines auditory instructions with visual stimuli to guide movement and enhance mental engagement.

## Data Availability

The dataset analyzed in the current study is available from the corresponding author on reasonable request.
